# Achromatic optical retardation from perovskites

**DOI:** 10.1038/s41377-021-00638-y

**Published:** 2021-11-04

**Authors:** Daniela Täuber

**Affiliations:** Leibniz Institute of Photonic Technology, and Friedrich-Schiller University, Abbe Center of Photonics and Institute of Physical Chemistry, Jena, Germany

**Keywords:** Photonic devices, Nanoparticles

*Nature Photonics* **15**, 813–816 (2021)

10.1038/s41566-021-00865-0

Control of light polarization over a wide range of optical wavelengths is highly desirable within a variety of applications. Fabrication of ‘waveplates’ allowing for achromatic retardation usually requires a sophisticated combination of multiple layers of birefringent materials. A team of researchers from the School of Materials Science & Engineering, the School of Physics and the School of Optics and Photonics at the Beijing Institute of Technology in China and from the Department of Chemistry at Princeton University in the United States, has found a very promising alternative approach using solution-processed ordered self-assembly of halide perovskites. Such fabricated birefringent Cs_4_PbBr_6_ crystal interspersed with CsPbBr_3_ nanocrystals showed good achromatic quarter-wave retardance over the spectral range of 532–800 nm. Further work on self-assembled birefringent materials with embedded nanocrystals is expected to provide access to inexpensive high quality achromatic waveplates for a wide field of applications ranging from opto-electronics to biomedical diagnostics.

